# Uncommon Cause of Paradoxical Embolism in a Case of Scimitar Syndrome

**DOI:** 10.1155/2013/352128

**Published:** 2013-12-21

**Authors:** Fortune O. Alabi, Fred Umeh, Maximo Lama, Francis G. Christian

**Affiliations:** Pulmonary Department, Florida Hospital Celebration Health, Celebration, FL 34747, USA

## Abstract

Scimitar syndrome, a rare congenital cardiopulmonary condition, presents in both pediatric and adult populations as an anomalous pulmonary venous return of most of the right lung to the inferior vena cava. Recently, asymptomatic adult cases have been diagnosed with advances in imaging studies. We report the case of an asymptomatic 43-year-old male, with a complex variant scimitar syndrome diagnosed by computed tomographic angiography.

## 1. Introduction

Scimitar syndrome is a partial anomalous venous return of the pulmonary vein to the inferior vena cava (IVC) rather than directly to the left atrium. There are associated right lung hypoplasia and in certain cases cardiac, diaphragmatic, vertebral, and genitor-urinary abnormalities [1]. Normal pulmonary venous anatomy results in the pulmonary veins draining the left and right lungs forming separate orifices in the posterior wall of the left atrium. When all pulmonary vessels drain directly into the right atrium, or a venous connection with the right atrium exists, this is designated total anomalous pulmonary venous return (TAPVR). Partial anomalous pulmonary venous return (PAPVR) is when one or more but not all pulmonary veins drain into the right atrium or a connecting vein. Scimitar syndrome is therefore a variant of PAPVR. Since the anomalous pulmonary vein connects with a systemic vein before returning to the right side of the heart, mixing of oxygenated blood with the deoxygenated systemic blood occurs.

Scimitar syndrome presentation varies based on the severity of clinical signs and symptoms. A high degree of left to right shunting along with cardiac and pulmonary defects is seen in symptomatic infants. They present with poor feeding, failure to thrive, and cyanosis. An isolated anomalous single pulmonary vein without congenital cardiac and pulmonary defects can present asymptomatically. The degree of left to right shunting is dependent on the number and size of anomalous veins as well as the pulmonary lobes from which the anomalous veins originate [2].

Diagnosis of the condition may be initially made by echocardiogram as a first step in symptomatic patients who present with cardiac physical exam findings [3]. In asymptomatic patients, the initial diagnosis may be suspected by an X-ray showing an unusually large anomalous vein paralleling the right heart border.

## 2. Case Presentation

**Figure 1:**
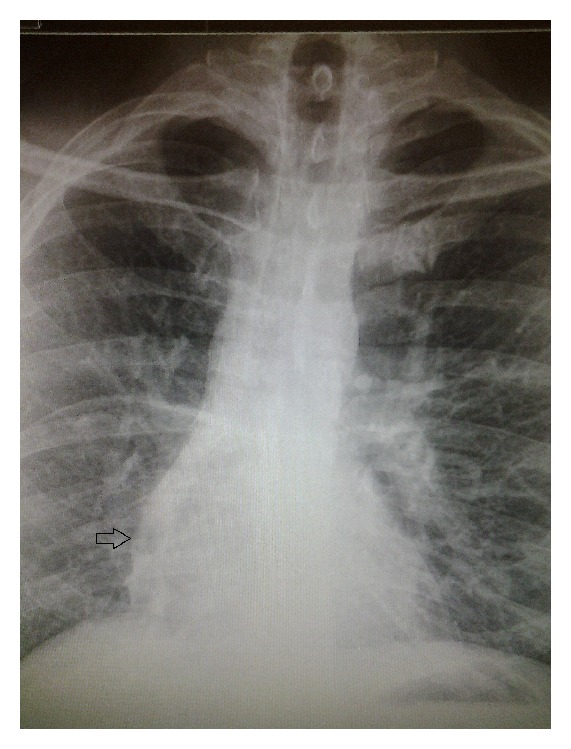
PA chest X-ray showing shadow representing anomalous pulmonary vein coursing alongside right heart border caudally.

**Figure 2:**
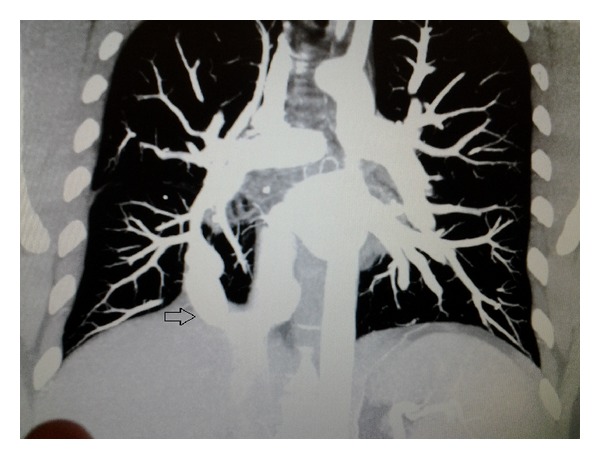
Coronal plane CTA showing anomalous vein drainage into suprahepatic IVC.

**Figure 3:**
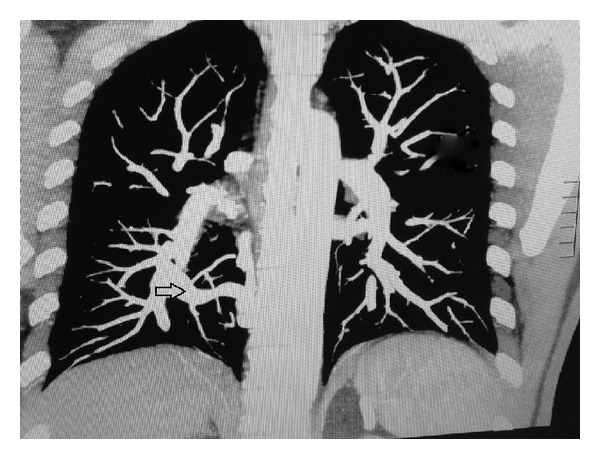
Coronal plane CTA showing fistulous connection between anomalous vein and right inferior pulmonary vein.

An otherwise healthy 43-year-old male, with no significant past medical history, presented to the pulmonary clinic for mild chest discomfort four weeks after being diagnosed with bronchitis at a walk in clinic. On physical exam, patient was active and pleasant with good air entry and clear breath sounds. There were normal cardiovascular findings with no murmurs noted. Imaging studies on presentation consisted of a PA chest X-ray positive for a left hilar density and a shadow alongside the right border of the heart (Figure 1). The chest X-ray was compared to a noncontrast CT performed two years earlier which showed a questionable arteriovenous malformation. Subsequent computed tomographic angiography (CTA) of the chest was performed to rule out arterio-venous fistula. Results of the CTA concluded a diagnosis of scimitar syndrome based on a partial anomalous pulmonary venous return of the right upper, middle, and lower lobes into the suprahepatic IVC (Figure 2) by way of a large anomalous scimitar vein. A fistulous connection between the large anomalous pulmonary vein and the right inferior pulmonary vein which drains into the left atrium was also noted (Figure 3). There is a congenital absence of the right superior pulmonary vein and corresponding hypoplastic right lung with mild dilation of the right atrium and right ventricle due to left to right shunting. A consultation with cardiothoracic surgery was obtained for cardiac catheterization to assess hemodynamic flow. Catheterization revealed anomalous pulmonary venous return from the right side, right ventricular and atrial enlargement, and significant left to right shunting with a Qp/Qs ratio of 2.1 to 1. Due to a potential pathway between the venous system and left atria, it was felt that the patient would benefit from surgical repair of the condition. This was performed using an anterolateral thoractomy approach. Utilizing cardiopulmonary bypass, the large scimitar vein was ligated, clipped, and anastomosed to the left atrium using a #16 Gore-tex interposition graft. The smaller anomalous vein draining as a fistula through the left atrium via the right inferior pulmonary vein was identified and left in place as with this correction it no longer provides a passage from the IVC to left atria. After surgery, the patient recovered without incident and is currently on Coumadin with target INR 2.0-3.0 for the next 6 months. Follow-up cardiac MRI revealed a patent interpositional graft with no significant gradient across the anastomosis and no left to right shunting, with Qp/Qs of 1.0.

## 3. Discussion

Scimitar syndrome describes a constellation of findings with the main finding being a PAPVR. The “scimitar” sign is the unique radiologic finding of a “tubular-shaped opacity extending towards the diaphragm along the right side of the heart” [4]. This shape of the anomalous vein as it appears on imaging studies resembles a Turkish sword, the “Pala” [5]. This rare congenital syndrome has a reported prevalence of 2 in 100,000 live births and 2 : 1 female preponderance with most diagnoses occurring in the pediatric age group [6].

Scimitar syndrome is classified based on clinical presentation as (a) infantile with significant symptoms and mortality or (b) child/adult with asymptomatic presentation commonly incidentally found in imaging studies. Furthermore, variants of the syndrome have been described. These include the Scimitar vein draining both into the IVC and directly into the left atrium and a variant with a “meandering” pulmonary vein coursing near to but not draining into the IVC [7]. The aforementioned patient presentation would fall under the classification of adult scimitar syndrome with unique variation. The fistulous connection between the anomalous vein and a normal coursing right inferior pulmonary vein provides an uncommon pathway for a paradoxical embolus depending on the direction of flow in the main scimitar vein.

Asymptomatic adult patients do not require surgical intervention as there is normal life expectancy without a clinically significant left to right shunt. Indications for surgery include left to right shunt >50%, resulting in pulmonary hypertension or heart failure, recurrent pulmonary infections, and anomalous vein compression of surrounding structures [2]. Advanced imaging should clearly map the course of the anomalous vein as well as associated connections as previous cases have reported scimitar syndrome with pulmonary arterio-venous fistulas giving rise to emboli causing neurologic defects [8]. Contrast-enhanced magnetic resonance angiography (CE-MRA) and computed tomographic angiography (CTA) are two commonly used imaging studies capable of fully characterizing and coursing the complex pulmonary venous connections encountered in PAPVR and its variants such as scimitar syndrome [9]. This case highlights scimitar syndrome with a fistulous connection between the scimitar and normal coursing inferior pulmonary vein. Surgical correction of the potential embolic pathway from this unique variant resulted in normal directed pulmonary flow and resolution of the associated left to right shunt.
